# Chloroquine inhibits Spring viremia of carp virus replication by modulating autophagy and metabolic pathways

**DOI:** 10.3389/fimmu.2026.1920055

**Published:** 2026-10-07

**Authors:** Yuchao Zhu, Shuyang Chen, Yue Wang, Jian Shen, Libo He, Yaping Wang, Dong Wang, Shuyan Miao, Guobin Chang, Pengfei Chu

**Affiliations:** 1 College of Animal Science and Technology, Yangzhou University, Yangzhou, China; 2 State Key Laboratory of Breeding Biotechnology and Sustainable Aquaculture, Institute of Hydrobiology, Chinese Academy of Sciences, Wuhan, China; 3 University of Chinese Academy of Sciences, Beijing, China; 4 Innovative Academy of Seed Design, Chinese Academy of Sciences, Beijing, China; 5 Yizheng Water World Agricultural Ecological Park Co., Ltd, Yangzhou, China

**Keywords:** autophagy, chloroquine, metabolomics, SVCV, transcriptomics

## Abstract

Spring viremia of carp virus (SVCV) severely threatens cyprinid aquaculture, yet the role of autophagy in SVCV infection remains unclear. Here, SVCV infection was associated with enhanced autophagic activity and autophagic flux in common carp and EPC cells. Pharmacological perturbation of autophagy revealed that rapamycin treatment increased SVCV gene expression and aggravated SVCV-induced cytopathic effects, whereas chloroquine blocked autophagic flux, suppressed viral gene expression, and mitigated virus-induced cellular damage. Transcriptomic analysis identified 1,868 differentially expressed genes after chloroquine treatment, with major changes in lysosome/autophagy-related pathways, glycan degradation, ion transport, cell cycle, DNA replication, and DNA repair. Metabolomic and integrated multi-omics analyses revealed changes in amino sugar and nucleotide sugar metabolism, steroid biosynthesis, riboflavin metabolism, and amino acid metabolism. Functional validation showed that diflorasone and all-trans-retinal reduced SVCV-G expression, alleviated cytopathic effects, and improved survival after SVCV infection. These findings suggest that SVCV infection is associated with enhanced autophagic flux, whereas chloroquine treatment blocks autophagic flux and is accompanied by reduced SVCV gene expression.

## Introduction

Spring viremia of carp virus (SVCV), a negative-sense single-stranded RNA virus belonging to the family Rhabdoviridae, is the causative agent of spring viremia of carp, a highly contagious and systemic viral disease in cyprinid fish (1, 2). SVCV mainly infects common carp (*Cyprinus carpio*) and other economically important freshwater fish, causing hemorrhage, ascites, exophthalmia, lethargy, and high mortality, especially in juvenile fish and under low-temperature conditions (3, 4). Owing to its broad host range, rapid transmission, and serious impacts on carp farming, SVCV remains a major viral pathogen threatening freshwater aquaculture. Although host immune responses to SVCV have been extensively characterized, the cellular processes and metabolic events that influence SVCV infection are still not fully elucidated.

Autophagy is an evolutionarily conserved intracellular degradation pathway that delivers damaged organelles, misfolded proteins, and invading pathogens to lysosomes for degradation. This cascade generally encompasses autophagosome biogenesis, autophagosome–lysosome fusion, and lysosomal degradation, collectively constituting complete autophagic flux (5, 6). During viral infection, autophagy exerts complex, context-dependent functions. In certain scenarios, autophagy contributes to antiviral defense by directly degrading viral components or regulating innate immune signaling. Nevertheless, many viruses have evolved strategies to hijack autophagic membranes, autophagy-related proteins, or autophagic flux to facilitate their replication and dissemination (7, 8). In aquatic organisms, autophagy has also been shown to participate in host responses to viral infection, yet its function varies across distinct virus–host systems (9–11). Accordingly, determining how autophagy and autophagic flux are associated with SVCV infection is important for understanding the interplay between SVCV and host cells.

Chloroquine is a lysosomotropic compound routinely employed to suppress the late phase of autophagy. It accumulates in acidic organelles and disrupts lysosomal function, thereby preventing autophagosome degradation and compromising autophagic flux (12). Because of this property, chloroquine has been widely used as a pharmacological tool to investigate the contribution of autophagic flux to viral infection. Nevertheless, chloroquine may also influence multiple cellular processes, including lysosomal activity, membrane trafficking, cell proliferation, DNA replication, and cellular metabolism. Accordingly, the effects of chloroquine on SVCV infection may not be attributable solely to autophagy inhibition and may also involve broader changes in host transcriptional and metabolic responses.

Host metabolism is closely linked to viral replication. Viruses depend on host-derived energy, nucleotides, amino acids, lipids, and membrane structures to complete their life cycles (13, 14). Changes in carbohydrate, lipid, amino acid, and nucleotide metabolism may therefore influence multiple stages of viral infection, including viral entry, genome replication, assembly, and release. Moreover, autophagy and lysosomal activity are closely connected with metabolic homeostasis, because they regulate nutrient recycling, organelle quality control, and intracellular material turnover. Therefore, investigating metabolic changes associated with autophagy modulation may provide new insight into the host responses associated with SVCV infection and antiviral defense (15). Multi-omics approaches provide effective tools for systematically analyzing host responses to viral infection and drug treatment. Transcriptomics reveals global changes in gene expression and biological pathways, while metabolomics identifies altered metabolites and metabolic pathways. Integrated transcriptomic and metabolomic analysis can further connect gene expression changes with metabolite alterations, thereby providing a more comprehensive view of host regulatory networks. Such approaches have been increasingly used in aquaculture research to identify key pathways involved in pathogen infection, immune regulation, and disease resistance (16, 17).

In the present study, we investigated the association between autophagic flux and SVCV infection using common carp and EPC cells. First, autophagy-related responses following SVCV infection were examined both *in vivo* and *in vitro*. Then, rapamycin and chloroquine were used to pharmacologically modulate autophagic flux and evaluate their effects on SVCV gene expression and virus-induced cytopathic effects. ATG5 knockdown was further performed to provide genetic evidence for the involvement of autophagy in SVCV infection. To characterize the host responses associated with chloroquine treatment, transcriptomic and metabolomic profiling were performed, followed by integrated multi-omics analysis. Finally, selected differential metabolites were functionally evaluated for their effects on SVCV infection. Overall, this study provides new insights into the interplay between autophagic flux, host transcriptional and metabolic remodeling, and SVCV infection, and provides a basis for further exploring autophagy- and metabolism-associated antiviral strategies against SVCV in aquaculture.

## Materials and methods

### Cells and virus

Epithelioma papulosum cyprini (EPC) cells were incubated at 28 °C in a humidified atmosphere with 5% CO_2_. The medium was supplemented with 10% fetal bovine serum and 1% Penicillin-Streptomycin. Spring viremia of carp virus (SVCV) was kindly provided by Professor Chao Pei of Henan Normal University and propagated in EPC cells. When typical cytopathic effects (CPEs) were observed, infected cells and culture supernatants were collected, subjected to repeated freeze–thaw cycles, and centrifuged to remove cell debris. The clarified viral supernatant was aliquoted and stored at −80 °C until further use.

### SVCV infection in fish and EPC cells

For infection experiment, a total of 300 common carp (average weight and length: 15 g and 10 cm) were intraperitoneally injected with SVCV or PBS, and the spleen tissues were collected at 5 days post-infection (dpi). The collected spleen tissues were subsequently subjected to ultrastructural observation via transmission electron microscopy. In addition, EPC cells were seeded into culture plates and infected with SVCV when the cell confluence reached approximately 70–80%. After viral adsorption for 3 h, the inoculum was discarded, and fresh culture medium was added. Cells were collected at 24 hpi for Western blot, qPCR, and LysoTracker staining.

### Transmission electron microscopy

Spleen tissues from control and SVCV-infected common carp were cut into small pieces and fixed with electron microscopy fixative. After fixation, samples were washed, post-fixed, dehydrated in a graded ethanol series, embedded in resin, and sectioned into ultrathin slices. The sections were stained with uranyl acetate and lead citrate and observed using a transmission electron microscope (Hitachi, HT7800). Autophagosome-like structures were identified based on their typical double-membrane morphology.

### Plasmid transfection and fluorescence observation

The GFP-LC3 plasmid was constructed by inserting the ORF sequence of LC3 into the pEGFP-N3 vector. To observe autophagosome formation, EPC cells were transfected with the GFP-LC3 plasmid using a transfection reagent according to the manufacturer’s instructions. After 12 h, cells were infected with SVCV for 24 h, fixed, and stained with DAPI. GFP-LC3 puncta were observed using a fluorescence microscope. To monitor autophagic flux, EPC cells were transfected with the tandem fluorescent reporter GFP-mCherry-LC3. After transfection, cells were infected with SVCV or treated with rapamycin or chloroquine. In this system, yellow puncta represent LC3-positive autophagosomes prior to fusion with lysosomes, whereas red puncta represent autolysosomes after lysosomal acidification. Fluorescence images were captured using a fluorescence microscope (Zeiss, LSM 880NLO).

For LysoTracker staining, EPC cells were seeded into culture plates and infected with SVCV. After 24 h, cells were incubated with LysoTracker Red according to the manufacturer’s protocol. Then, cells were washed with PBS and counterstained with DAPI. Lysosomal fluorescence signals were observed using a fluorescence microscope.

### Quantitative real-time PCR and Western blot analysis

Total RNA was extracted from EPC cells using an RNA extraction reagent according to the manufacturer’s instructions (Tiangen). RNA concentration and purity were measured using a spectrophotometer (Implen, NanoPhotometer^®^ N30). The RNA was reverse-transcribed into cDNA using a reverse transcription kit (Vazyme). Quantitative real-time PCR was performed using SYBR Green qPCR mix on a real-time PCR system (Applied Biosystems, 7500). The qPCR reaction system contained 20 μL SYBR Green Mixture, 10 μL Mix, 1 μL cDNA template, 1 μL each primer, and 7 μL deionized water. The thermal conditions were as follows: 95 °C for 5 min, 40 cycles of 95 °C for 20 s, 60 °C for 20 s, and 72 °C for 20 s. The relative expression levels were calculated using the 2^−ΔΔCt^ method, with β-actin used as the internal reference gene. All primers for the RT-qPCR assays are listed in **Supplementary Table S1**.

For Western blot analysis, cells were lysed in RIPA buffer containing protease inhibitors on ice for 30 min and protein extracts were separated using 12% SDS-PAGE and transferred onto the PVDF membranes. Then the membranes were blocked with 5% non-fat milk powder at room temperature for 1 h and incubated with primary antibody against LC3B (1:1000, Sigma), p62 (1:1000, Huaan), and β-actin (1:2000, Huaan) at 4 °C for 12 h. After the final wash, PVDF membranes were incubated with a secondary antibody at room temperature for 2 h. Finally, the immunoblot signals were detected using an enhanced chemiluminescence (ECL) Detection Kit (Tiangen).

### Crystal violet staining assay

Crystal violet staining was performed to assess the inhibitory effects of tested drugs on SVCV-induced CPEs. EPC cells were seeded in 6-well plates and cultured at 28 °C to 70–80% confluence. Chloroquine and rapamycin were both dissolved in DMSO at a concentration of 10 mM before use. Cells were pretreated with different concentrations of the indicated drugs (Chloroquine: 20 μM, rapamycin: 50 nM) for 12 h. Subsequently, SVCV serially diluted in serum-free medium was added for 3 h of viral adsorption at 28 °C. After incubation, the viral inoculum was discarded, and cells were gently rinsed with PBS to eliminate unbound virions. Fresh complete medium was replenished for continuous culture. At 48–72 h post-infection, obvious CPEs (including cell rounding, shrinkage, detachment, and monolayer disruption) were observed in the virus control group. The culture medium was removed, and cells were rinsed with PBS, followed by fixation with 4% paraformaldehyde for 15–20 min at room temperature. Fixed cells were stained with 0.5% crystal violet solution for 15–20 min at room temperature. Excess dye was washed off with distilled water, and the plates were naturally air-dried for subsequent observation.

### Transcriptomic, metabolomic, and integrated multi-omics analyses

EPC cells were cultured to approximately 70–80% confluence and pretreated with chloroquine (20 μM) or DMSO for 12 h. The cells were then infected with SVCV for 3 h to allow viral adsorption. After adsorption, the viral inoculum was removed, and the cells were gently washed with PBS and maintained in fresh culture medium. At 24 hpi, cells from the control and chloroquine-treated groups were collected for transcriptomic and metabolomic analyses. Three independent biological replicates were prepared for each experimental group and subjected to RNA sequencing (RNA-seq) and untargeted metabolomic profiling, respectively.

For transcriptome sequencing, total RNA was isolated, quality-checked, and used for library construction. The libraries were sequenced on an Illumina platform. Raw reads (NCBI BioProject accession: PRJNA1482244) were filtered to remove adapter sequences and low-quality reads, and clean reads were used for subsequent analysis. Sequencing quality was evaluated based on clean bases, Q20, Q30, and GC content. Principal component analysis (PCA) was performed to assess sample distribution. Differentially expressed genes (DEGs) between the control and chloroquine-treated groups were identified, followed by GO, KEGG, GSEA, and heatmap analyses.

For metabolomic analysis, metabolites were extracted from EPC cell samples and profiled by LC-MS/MS. Quality control samples were generated by pooling equal aliquots of all sample extracts to monitor data stability and reproducibility. Raw metabolomic data were processed for peak detection, alignment, normalization, and metabolite annotation. PCA was used to assess sample distribution, and differential metabolites were screened based on fold change, statistical significance, and multivariate analysis parameters. KEGG annotation and enrichment analyses were performed to identify significantly perturbed metabolic pathways.

Correlation analysis was performed to evaluate associations among differential metabolites and between DEGs and differential metabolites. Spearman correlation coefficients were calculated, and correlation heatmaps and networks were generated. Joint KEGG enrichment analysis was further conducted to identify pathways simultaneously enriched by DEGs and differential metabolites after chloroquine treatment.

### Validation of selected differential metabolites

Based on the metabolomic data, diflorasone and all-trans-retinal were selected for further validation. EPC cells were treated with graded concentrations of diflorasone or all-trans-retinal for 12 h, prior to SVCV infection. After 24 h, cells were collected for qPCR detection of SVCV-G transcript levels. In addition, crystal violet staining was performed to evaluate SVCV-induced cytopathic effects. Briefly, infected cells were fixed and stained with crystal violet solution. After washing and drying, images were captured to assess cell monolayer integrity and cytopathic damage.

For the survival assay, one-month-old zebrafish (n = 40 per group) were divided into 6 groups, including a blank control group, an SVCV control group, two diflorasone-treated groups (0.01 μM and 0.05 μM), and two all-trans-retinal-treated groups (0.1 μM and 0.5 μM). Zebrafish were administered 10 μL of PBS or the indicated compounds by gavage every other day for one week. After the treatment period, zebrafish were challenged with SVCV by oral gavage. Mortality was recorded daily for 14 consecutive days, and the accumulated survival rate was calculated.

### RNAi experiments

To further investigate the role of autophagy during SVCV infection, EPC cells were transfected with siRNAs targeting ATG5 or negative control by using lipofectamine3000 (Thermo Fisher Scientific). After transfection for 24 h, cells were infected with SVCV and harvested at 24 h post-infection. The harvested cells were used for further analysis. The siRNAs sequences were shown in **Supplementary Table S1**.

### Statistical analysis

All experiments were performed at least three times (n ≥ 3). Experimental data are expressed as the mean ± SD. Statistical differences among groups were assessed by one-way analysis of variance (ANOVA), followed by Dunnett’s multiple comparisons test for comparisons of each treatment group against the control group.

## Results

### SVCV infection activates autophagy in common carp and EPC cells

Autophagy is intricately involved in viral infection and replication in aquatic animals (18). In this study, autophagy was markedly activated in the spleen of common carp following SVCV infection, as evidenced by an increased number of autophagosomes (**Figure 1A**). LC3-I is conjugated to phosphatidylethanolamine to form LC3-II, which is then recruited to autophagosomal membranes and serves as a well-established marker of autophagy. In EPC cells, LC3-II-labeled autophagosomes were observed as prominent green puncta following SVCV infection, whereas only faint punctate signals were observed in the control group (**Figure 1B**). Consistently, the increased conversion of LC3-I to LC3-II, accompanied by a decrease in p62 protein levels, suggested enhanced autophagic activity in the SVCV-infected group (**Figure 1C**). In line with these observations, lysosomal accumulation was also enhanced following SVCV infection, as indicated by the enhanced red fluorescent signal (**Figure 1D**). Collectively, these findings indicate that SVCV infection activates autophagy both *in vivo* and *in vitro*.

**Figure 1:**
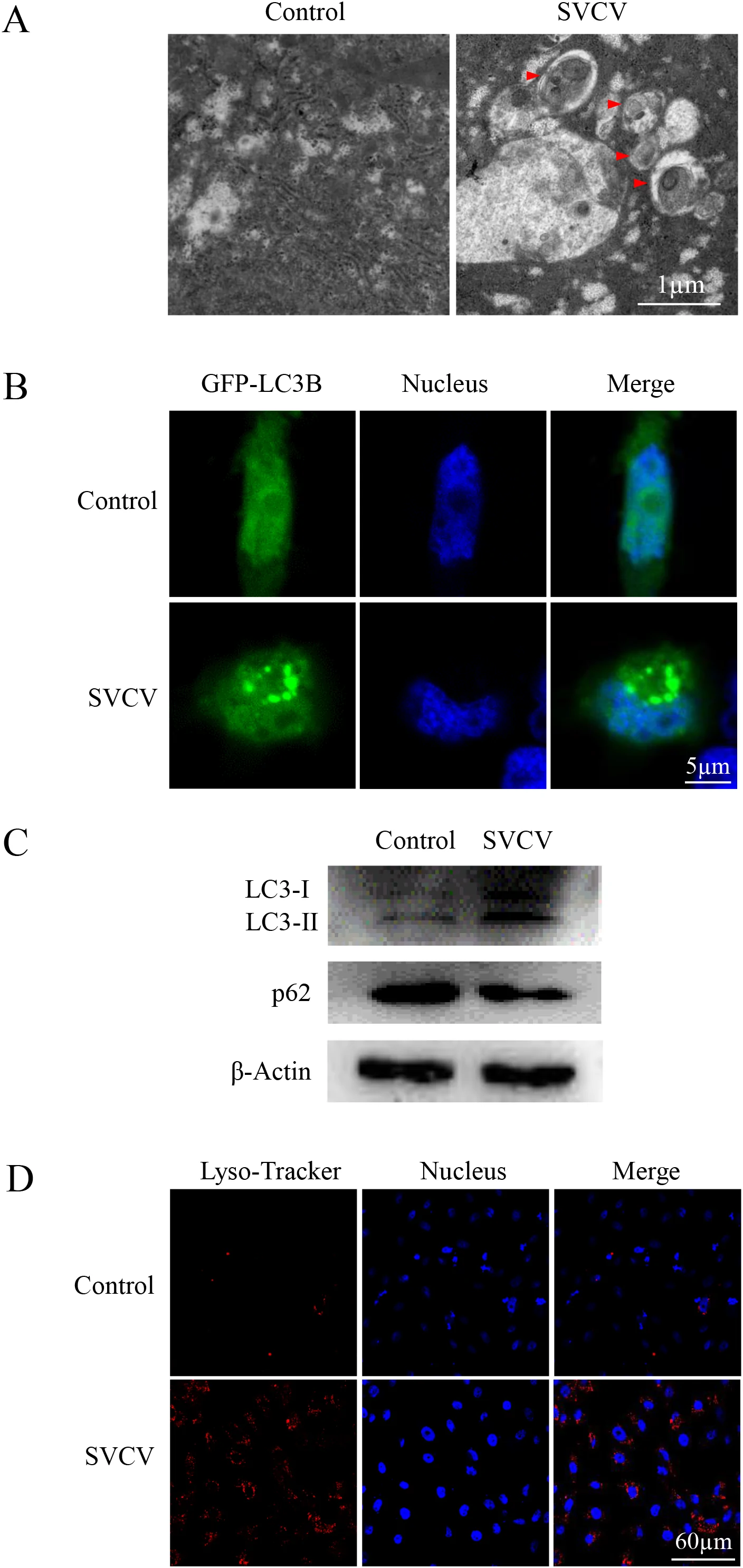
SVCV infection induces autophagy in common carp and EPC cells. **(A)** Transmission electron microscopy analysis of autophagic structures in the spleen of common carp following SVCV infection. Red arrows indicate representative autophagosomes. **(B)** EPC cells were transfected with the GFP-LC3 plasmid and subsequently infected with SVCV. GFP-LC3 puncta were visualized by fluorescence microscopy. Green puncta indicate GFP-LC3-labeled autophagosomes, and nuclei were stained with DAPI. **(C)** Western blot analysis of autophagy-related proteins LC3 and p62 in EPC cells following SVCV infection. β-actin was used as the internal control. **(D)** LysoTracker staining of lysosomes in EPC cells following SVCV infection. Red fluorescence indicates LysoTracker-positive lysosomal signals, and nuclei were counterstained with DAPI.

### Modulation of autophagic flux affects SVCV replication

Autophagosome–lysosome fusion is a critical step in maintaining functional autophagic flux. To further determine whether SVCV infection regulates this process, EPC cells were transfected with the tandem fluorescent GFP-mCherry-LC3 reporter. In this system, LC3-positive autophagosomes exhibit both GFP and mCherry fluorescence and therefore appear as yellow puncta. Upon autophagosome–lysosome fusion, the acidic lysosomal lumen quenches the GFP signal, whereas mCherry fluorescence remains stable. Thus, red puncta represent mature autolysosomes. Compared with the uninfected control, SVCV infection markedly increased the number of red puncta, exhibiting a phenotype similar to that induced by the classical autophagy activator rapamycin. In contrast, chloroquine treatment resulted in a pronounced accumulation of yellow puncta, indicating impaired lysosomal acidification and blocked autophagic flux (**Figure 2A**). Collectively, these results indicate that SVCV infection promotes autophagic flux in EPC cells.

**Figure 2:**
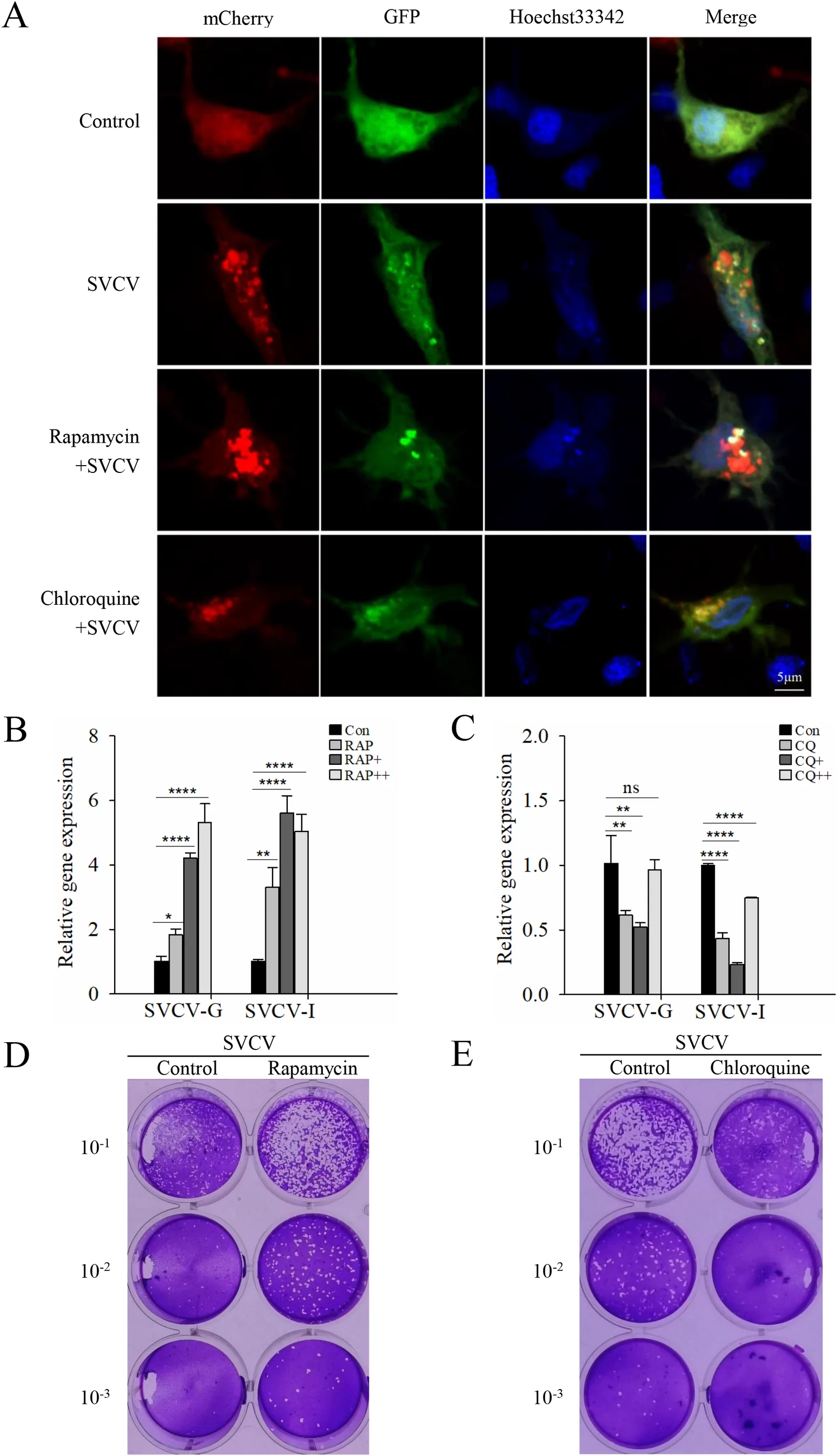
Modulation of autophagic flux affects SVCV replication in EPC cells. **(A)** EPC cells were transfected with the GFP-mCherry-LC3 reporter, followed by SVCV infection with or without rapamycin or chloroquine treatment. Yellow puncta represent LC3-positive autophagosomes, and red puncta represent autolysosomes. **(B, C)** qPCR quantification of SVCV-G and SVCV-I transcript levels in SVCV-infected EPC cells upon rapamycin or chloroquine treatment. Con: untreated control, RAP: (25 nM), RAP+: (50 nM), and RAP++: (100 nM) represent SVCV-infected cells treated with different concentrations of rapamycin; CQ: (10 μM), CQ+: (20 μM), and CQ++: (40 μM) represent SVCV-infected cells treated with different concentrations of chloroquine. **(D, E)** Crystal violet staining displaying SVCV-triggered cytopathic effects in SVCV-infected EPC cells with the indicated drug treatments (rapamycin: 50 nM, chloroquine: 20 μM). Data are presented as mean ± SD of three independent biological replicates. One-way ANOVA followed by Dunnett’s multiple comparisons test was performed. *P < 0.05, **P < 0.01, ****P < 0.0001.

We next explored the functional relationship between autophagic flux and SVCV replication. To pharmacologically modulate autophagy, SVCV-infected EPC cells were treated with the autophagy activator rapamycin or the autophagic flux inhibitor chloroquine. qPCR analysis showed that rapamycin treatment significantly increased the mRNA expression levels of SVCV-G and SVCV-I. In contrast, chloroquine treatment markedly suppressed the transcription of both viral genes (**Figures 2B, C**). Consistent with these transcriptional changes, crystal violet staining further verified that rapamycin aggravated SVCV-induced cytopathic damage, whereas chloroquine effectively alleviated SVCV-induced CPE in EPC cells (**Figures 2D, E**).

To further genetically validate the role of autophagy in supporting SVCV replication, we knocked down ATG5, an essential core factor involved in autophagosome formation, via siRNA transfection. Efficient silencing of ATG5 at 24 h and 36 h post-transfection was validated by qPCR (**Figure 3A**). Upon SVCV challenge, CPEs were less pronounced, and the transcript levels of SVCV-G and SVCV-I were significantly lower in ATG5-depleted cells than in control cells at both time points (**Figures 3B–D**). Consistent with the pharmacological results, ATG5 knockdown further supported a positive association between autophagy and SVCV replication. Taken together, these findings suggest that pharmacological activation of autophagy by rapamycin favors SVCV replication, whereas disruption of autophagy by chloroquine treatment or ATG5 knockdown restricts viral replication in EPC cells.

**Figure 3:**
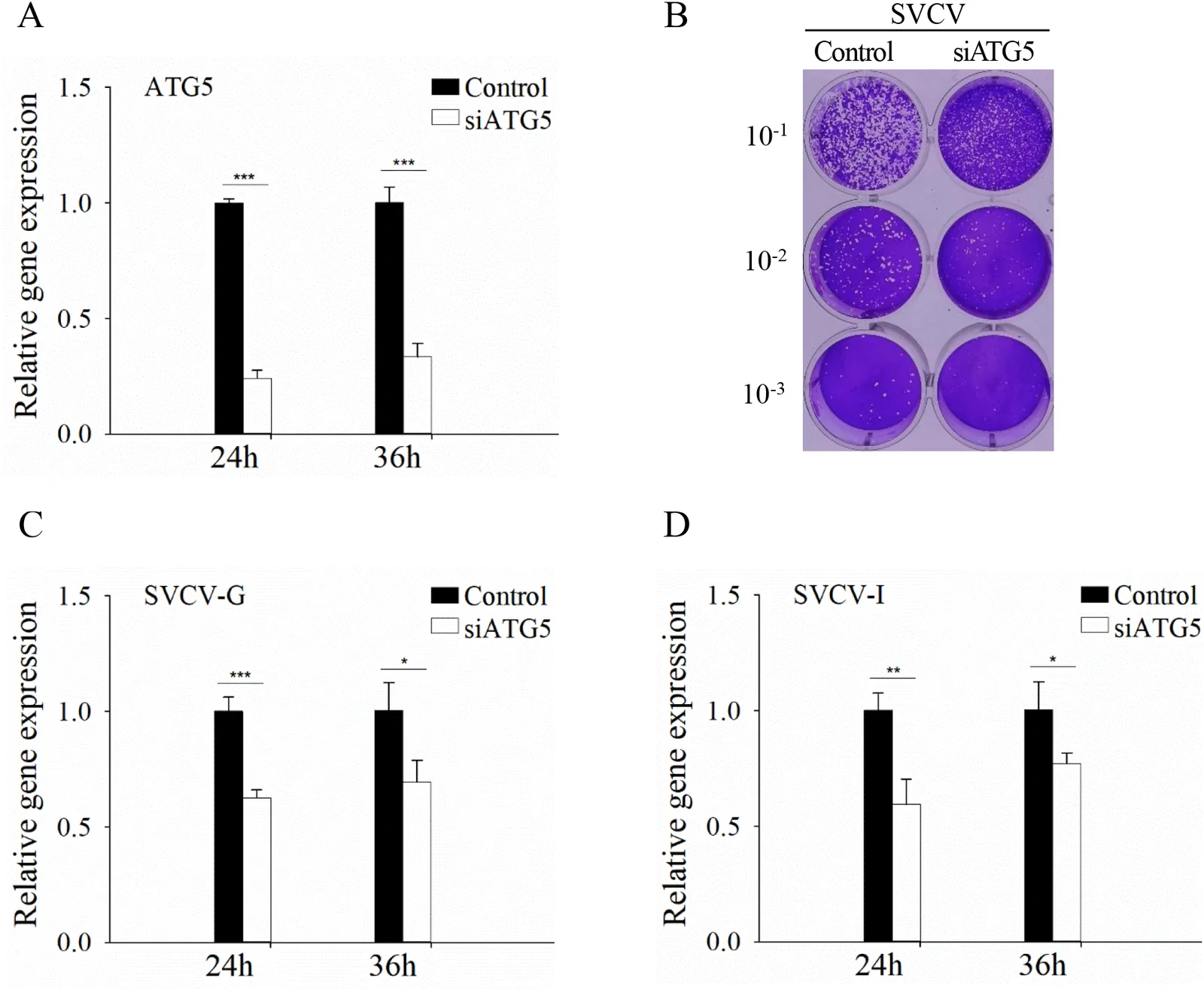
ATG5 knockdown suppresses SVCV infection in EPC cells. EPC cells were transfected with ATG5-specific siRNA or negative control siRNA. **(A)** The knockdown efficiency of ATG5 was evaluated by qPCR at 24 and 36 h post-transfection. **(B)** Crystal violet staining was performed to assess the function of ATG5 on SVCV-induced cytopathic effects. **(C, D)** Relative mRNA expression levels of SVCV-G **(C)** and SVCV-I **(D)** in ATG5-depleted and control cells following SVCV infection. Data are presented as the mean ± SD. Statistical significance was determined by comparison with the corresponding control group. *P < 0.05, **P < 0.01, ***P < 0.001, ****P < 0.0001.

### Overview of transcriptome sequencing and differential expression analysis

To investigate the potential molecular mechanisms underlying the antiviral effect of chloroquine, transcriptome sequencing was performed in EPC cells. The RNA-seq data exhibited consistently high sequencing quality across all samples, with clean bases ranging from 6.07 to 6.53 Gb. The Q20 and Q30 values exceeded 97.9% and 94.1%, respectively, indicating high base-calling accuracy and low sequencing error rates. In addition, the GC content remained comparable among samples, demonstrating the overall stability and reliability of the sequencing data for subsequent transcriptomic analyses (**Supplementary Table S2**).

Principal component analysis (PCA) revealed a clear separation between the control group (Ct) and the chloroquine-treated group (Tt), indicating substantial differences in their global transcriptomic profiles (**Figure 4A**). Differential expression analysis identified a total of 1,868 DEGs between the Tt and Ct groups, including 1,061 upregulated and 807 downregulated genes (**Figure 4B**). The volcano plot further illustrated the overall distribution and magnitude of gene expression changes induced by chloroquine treatment (**Figure 4C**). Venn analysis showed that 14,044 expressed genes were shared between the Ct and Tt groups, while a subset of genes exhibiting group-specific expression patterns were also observed (**Figure 4D**). Collectively, these results indicate that chloroquine treatment induces substantial transcriptomic alterations in EPC cells, providing a basis for further functional enrichment and pathway analyses to identify molecular processes potentially associated with its antiviral activity.

**Figure 4:**
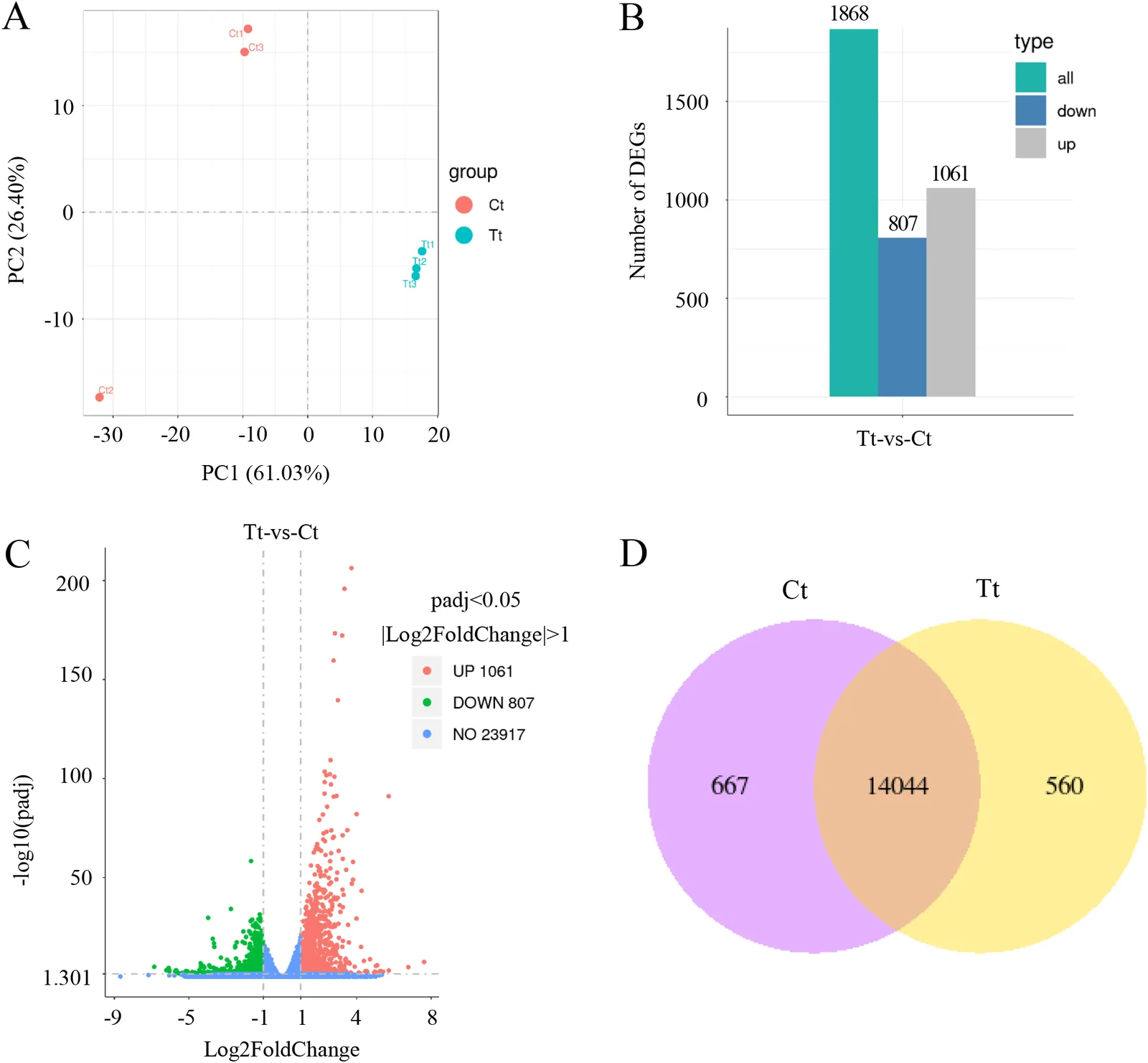
Overview of transcriptome sequencing and differential expression analysis following chloroquine treatment. **(A)** Principal component analysis (PCA) of RNA-seq data from the control (Ct) and chloroquine-treated (Tt) groups. **(B)** Statistical summary of differentially expressed genes (DEGs) between the Tt and Ct groups. **(C)** Volcano plot showing the distribution of DEGs between the Tt and Ct groups. **(D)** Venn diagram showing the number of shared and group-specific expressed genes between the Ct and Tt groups.

### Functional enrichment analysis of DEGs after chloroquine treatment

GO enrichment analysis showed that the upregulated DEGs were mainly enriched in terms associated with carbohydrate metabolic process, hydrolase activity acting on glycosyl bonds, ion transmembrane transport, proton transmembrane transport, cation transmembrane transport, and proton-transporting V-type ATPase complex (**Figure 5A**). The GSEA plots and heatmaps showed that genes related to glycan metabolism, hydrolase activity, ATPase complexes, and ion transport were generally expressed at higher levels in the chloroquine-treated group than in the control group (**Figures 5B–D**). In contrast, the downregulated DEGs were mainly enriched in chromosome organization, DNA metabolic process, DNA replication, organelle organization, microtubule binding, microtubule motor activity, and chromosomal region (**Figure 5E**). Consistently, the heatmaps showed reduced expression of genes involved in DNA replication, chromosome organization, and microtubule-associated processes in the chloroquine-treated group (**Figures 5F–H**).

**Figure 5:**
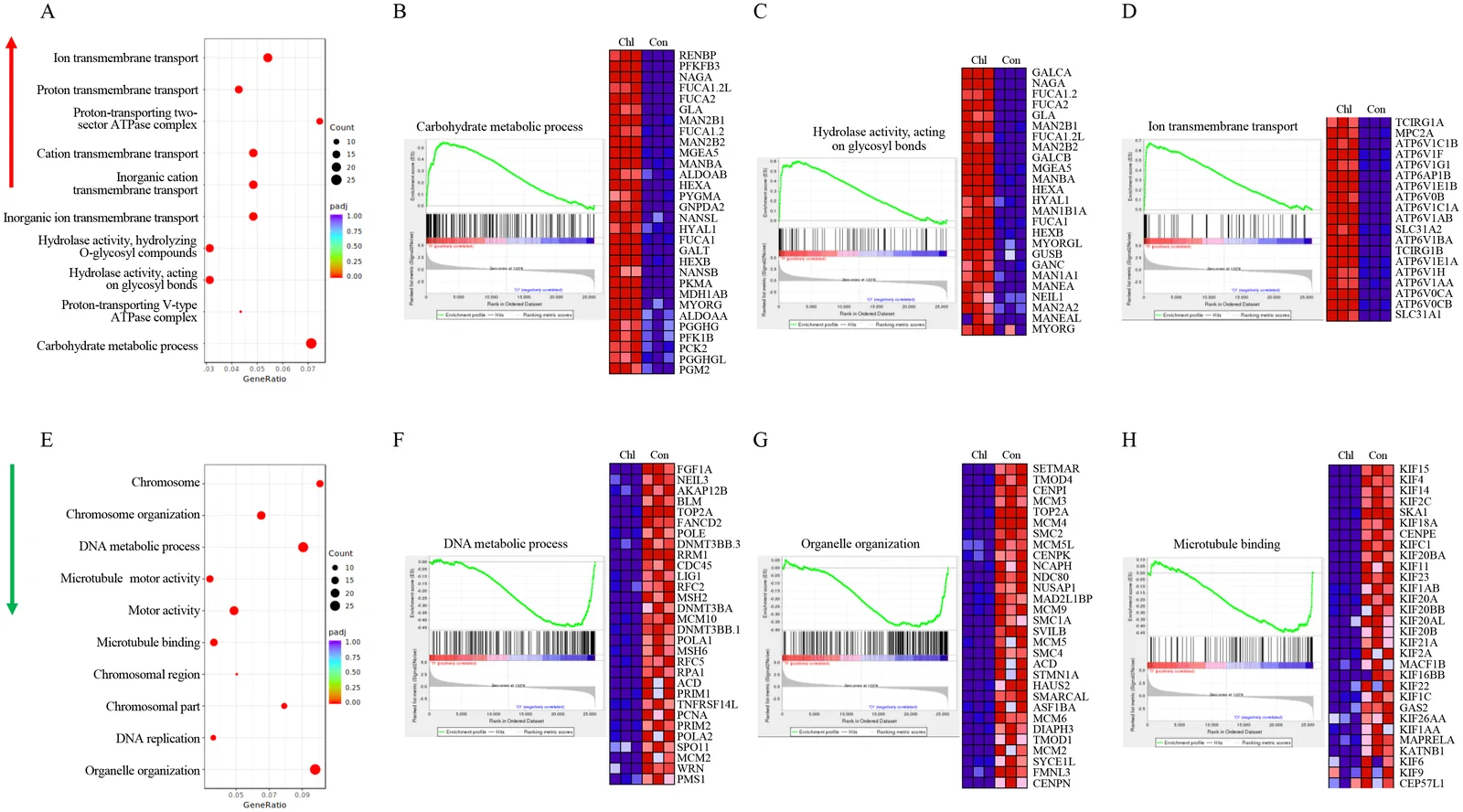
GO enrichment analysis of DEGs following chloroquine treatment. **(A)** GO enrichment analysis of upregulated DEGs in the chloroquine-treated group compared with the control group. **(B–D)** Representative enrichment plots and heatmaps of GO terms associated with upregulated DEGs, including carbohydrate metabolic process, hydrolase activity, acting on glycosyl bonds, and ion transmembrane transport. **(E)** GO enrichment analysis of downregulated DEGs in the chloroquine-treated group compared with the control group. **(F–H)** Representative enrichment plots and heatmaps of GO terms associated with downregulated DEGs, including DNA metabolic process, Organelle organization, and Microtubule binding.

KEGG enrichment analysis revealed distinct pathway profiles for the upregulated and downregulated DEGs. The upregulated DEGs were mainly enriched in pathways including lysosome, phagosome, autophagy–animal, glycosaminoglycan degradation, other glycan degradation, sphingolipid metabolism, oxidative phosphorylation, and mTOR signaling pathway (**Figures 6A–D**). In contrast, the downregulated DEGs were mainly enriched in cell cycle, DNA replication, Fanconi anemia pathway, homologous recombination, mismatch repair, p53 signaling pathway, and pyrimidine metabolism (**Figures 6E–H**). Collectively, these enrichment patterns indicate that chloroquine treatment is associated with substantial alterations in lysosome- and autophagy-related processes, cellular metabolism and related signaling pathways, accompanied by suppression of pathways involved in DNA replication and cell-cycle progression.

**Figure 6:**
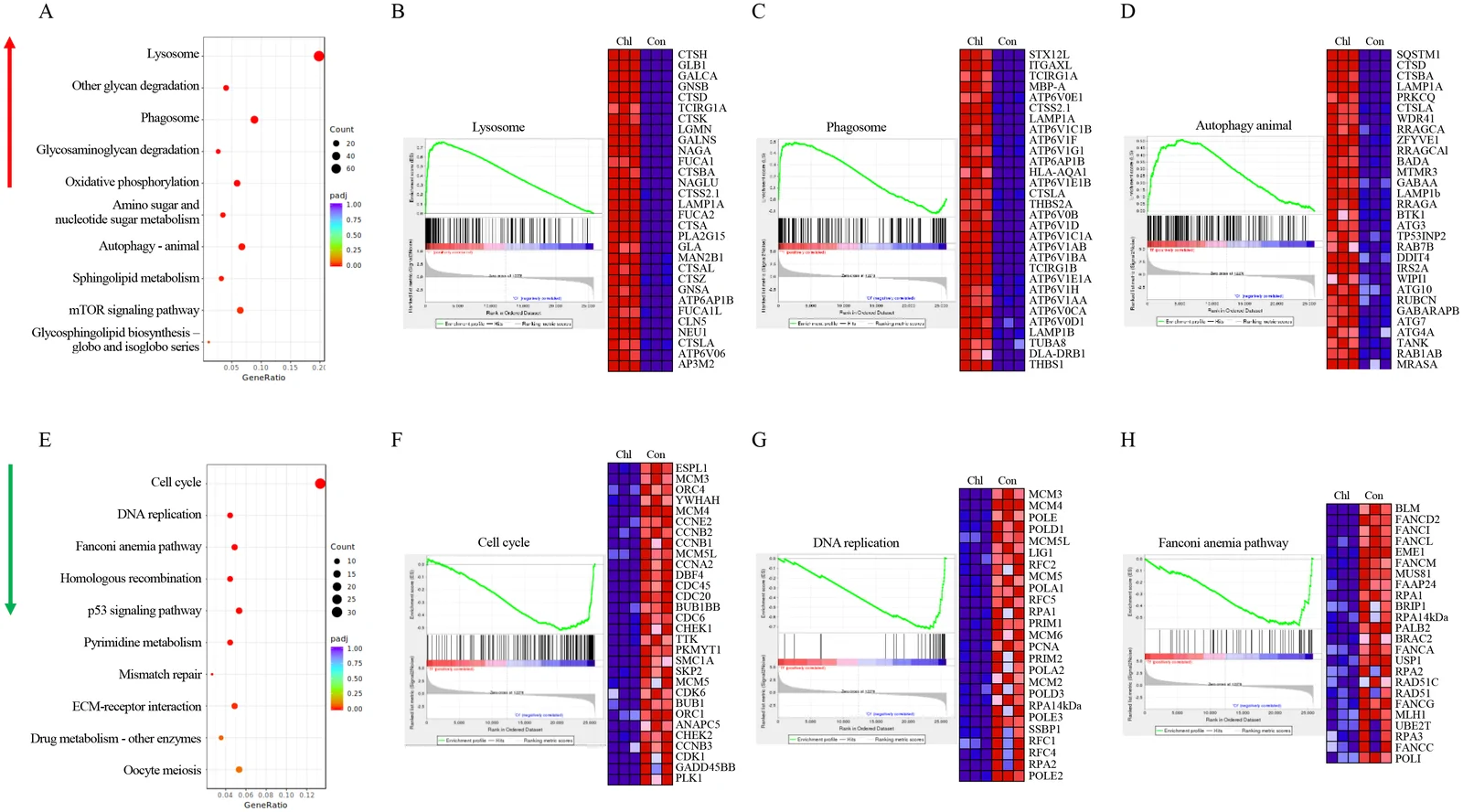
KEGG enrichment analysis of DEGs following chloroquine treatment. **(A)** KEGG enrichment analysis of upregulated DEGs in the chloroquine-treated group compared with the control group. **(B–D)** Representative enrichment plots and heatmaps of pathways associated with upregulated DEGs, including lysosome, phagosome, and autophagy–animal. Upregulated DEGs were mainly enriched in pathways associated with lysosome, phagosome, autophagy, glycan degradation, sphingolipid metabolism, oxidative phosphorylation, and mTOR signaling. **(E)** KEGG enrichment analysis of downregulated DEGs in the chloroquine-treated group compared with the control group. **(F–H)** Representative enrichment plots and heatmaps of pathways associated with downregulated DEGs, including cell cycle, DNA replication, and Fanconi anemia pathway. Downregulated DEGs were mainly enriched in pathways associated with the cell cycle, DNA replication, DNA repair, p53 signaling, and pyrimidine metabolism.

### Overview of metabolomic profiling after chloroquine treatment

To further investigate the potential metabolic mechanisms underlying the antiviral effect of chloroquine, metabolomic analysis was performed in control and chloroquine-treated cells. PCA analysis showed a clear separation between the control and chloroquine-treated groups, indicating substantial differences in their overall metabolic profiles following chloroquine treatment. The three biological replicates within each group clustered closely, demonstrating good reproducibility and stability of the metabolomic data (**Figure 7A**).

**Figure 7:**
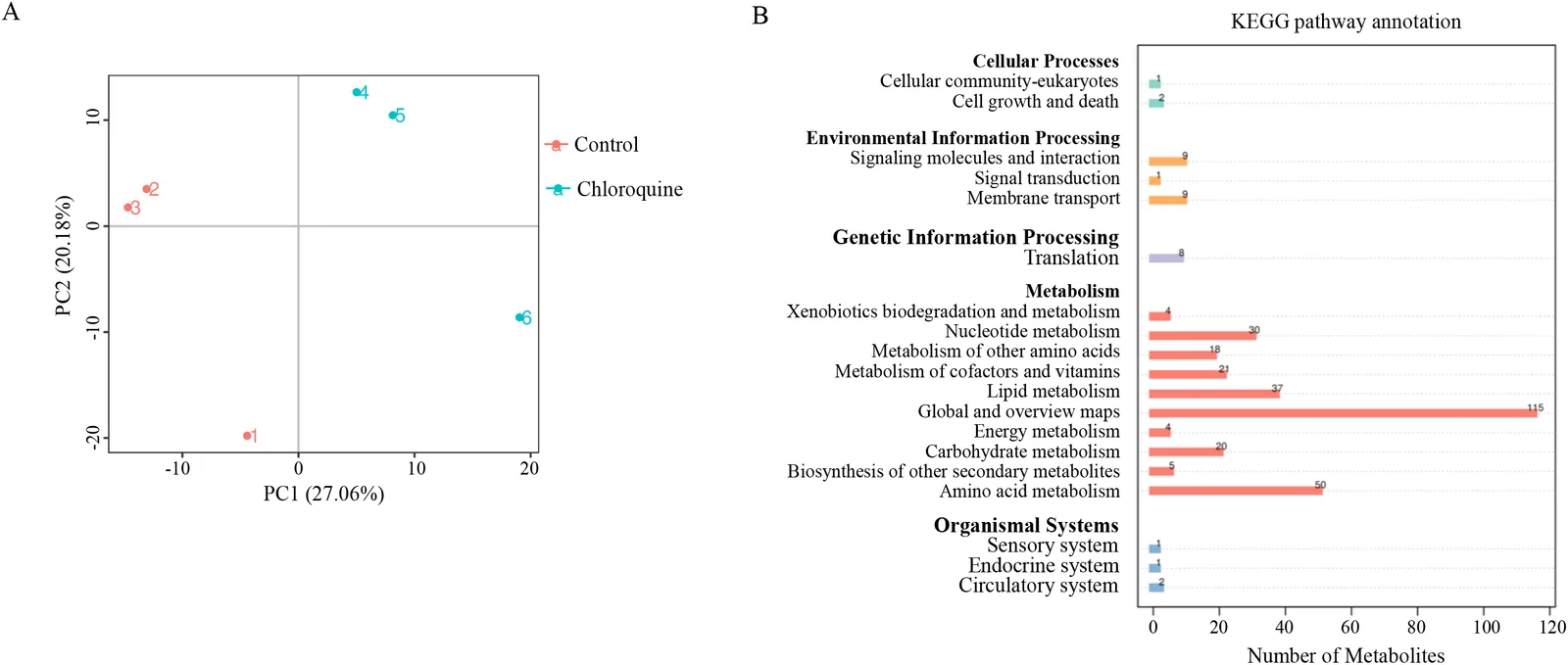
Overview of metabolomic profiling following chloroquine treatment. **(A)** PCA analysis of metabolomic data from the control and chloroquine-treated groups. **(B)** KEGG pathway classification of the identified metabolites. The number of metabolites assigned to each pathway category is shown on the x-axis.

KEGG pathway annotation showed that the identified metabolites were predominantly classified into metabolism-related pathways. Among these categories, global and overview maps contained the largest number of annotated metabolites (50 metabolites), followed by amino acid metabolism (50 metabolites), lipid metabolism (37 metabolites), and nucleotide metabolism (30 metabolites) (**Figure 7B**). These results indicate that chloroquine treatment is associated with broad metabolic alterations, providing a basis for subsequent identification of differential metabolites and pathway enrichment analysis.

### Enrichment analysis of differential metabolites

The heatmap revealed distinct abundance patterns of differential metabolites between the chloroquine-treated and control groups. Several metabolites, including PE (18:2e/22:4), 4-guanidinobutyric acid, propionyl-L-carnitine, 2-methylbutyrylcarnitine, glycylproline, dihydrouracil, UDP-N-acetyl-alpha-D-glucosamine, and benzoylecgonine, exhibited lower relative abundance in the chloroquine-treated group. In contrast, metabolites such as ergosterol, LysoPG 18:1, D-glucosamine 6-phosphate, QNK, nervonic ceramide, riboflavin, all-trans-retinal, and RNH were relatively more abundant following chloroquine treatment (**Figure 8A**).

**Figure 8:**
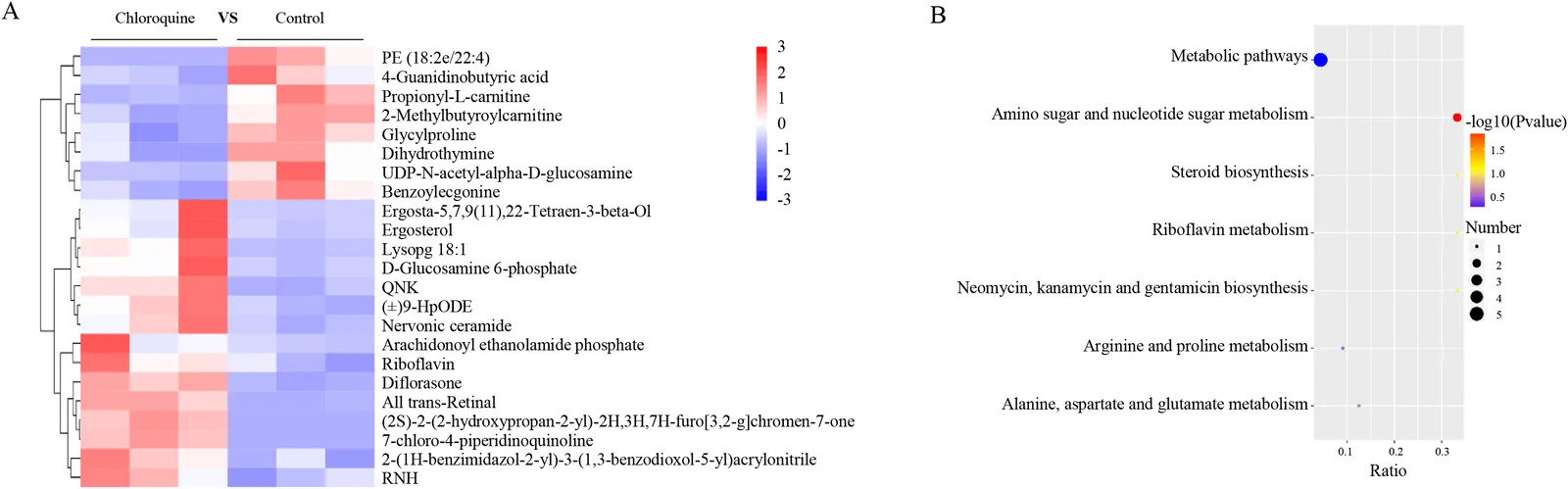
Differential metabolite analysis following chloroquine treatment. **(A)** Heatmap showing the relative abundance patterns of differential metabolites between the control and chloroquine-treated groups. **(B)** KEGG enrichment analysis of differential metabolites following chloroquine treatment. The dot size represents the number of differential metabolites enriched in each pathway, and the color indicates the enrichment significance.

KEGG enrichment analysis showed that the differential metabolites were mainly enriched in metabolic pathways, amino sugar and nucleotide sugar metabolism, steroid biosynthesis, riboflavin metabolism, arginine and proline metabolism, and alanine, aspartate and glutamate metabolism. Among these pathways, metabolic pathways contained the largest number of differential metabolites, while amino sugar and nucleotide sugar metabolism showed a relatively high enrichment ratio (**Figure 8B**). These results showed that the differential metabolites were mainly distributed in pathways related to sugar derivative metabolism, steroid biosynthesis, vitamin metabolism, and amino acid metabolism.

### Correlation analysis of differential metabolites

Correlation analysis was performed to evaluate the relationships among differential metabolites identified between the chloroquine-treated and control groups. The correlation network revealed extensive associations among these metabolites, with both positive and negative correlations observed. Several metabolites, including all-trans-retinal, diflorasone, PE (18:2e/22:4), propionyl-L-carnitine, UDP-N-acetyl-alpha-D-glucosamine, D-glucosamine 6-phosphate, ergosterol, riboflavin, and nervonic ceramide, showed multiple connections within the correlation network (**Figure 9A**).

**Figure 9:**
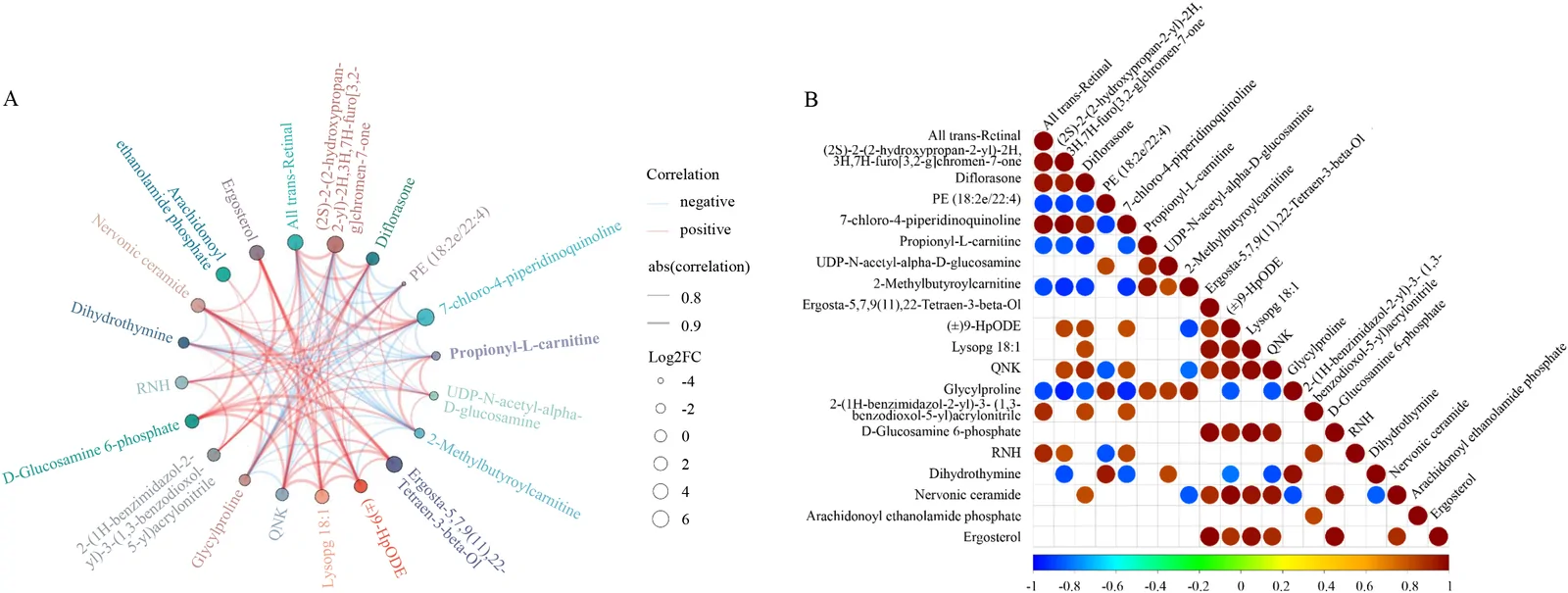
Correlation analysis of differential metabolites following chloroquine treatment. **(A)** Correlation network showing associations among differential metabolites identified between the control and chloroquine-treated groups. Red lines indicate positive correlations, whereas blue lines indicate negative correlations. **(B)** Correlation heatmap showing pairwise correlations among differential metabolites. Red represents positive correlations, and blue represents negative correlations.

The correlation matrix further revealed distinct association patterns among the differential metabolites. Some metabolites, such as ergosterol, D-glucosamine 6-phosphate, QNK, and LysoPG 18:1, were strongly positively correlated with one another, whereas metabolites including PE (18:2e/22:4), propionyl-L-carnitine, 2-methylbutyrylcarnitine, and glycylproline were negatively correlated with several metabolites that were increased following chloroquine treatment (**Figure 9B**). Collectively, these results indicate coordinated metabolic changes following chloroquine treatment and suggest potential functional links among multiple altered metabolites.

### Integrated transcriptomic and metabolomic analysis

Integrated transcriptomic and metabolomic analyses were performed to investigate the associations between differentially expressed genes (DEGs) and differential metabolites following chloroquine treatment. The correlation heatmap revealed extensive correlations between DEGs and differential metabolites, with several metabolites, including D-glucosamine 6-phosphate, UDP-N-acetyl-alpha-D-glucosamine, riboflavin, nervonic ceramide, propionyl-L-carnitine, and PE (18:2e/22:4), showing strong correlations with multiple DEGs (**Figure 10A**). These metabolites were mainly associated with amino sugar and nucleotide sugar metabolism, lipid metabolism, and vitamin-related metabolism. The correlation network further identified extensive positive and negative correlations between DEGs and differential metabolites, with PE (18:2e/22:4), diflorasone, all-trans-retinal, 7-chloro-4-piperidinoquinoline, and (2S)-2-(2-hydroxypropan-2-yl)-2H,3H,7H-furo[3,2-g]chromen-7-one showing associations with multiple DEGs (**Figure 10B**). Collectively, these findings indicate that chloroquine treatment is associated with coordinated alterations in transcriptional and metabolic profiles and suggest potential links between gene expression changes and metabolic remodeling.

**Figure 10:**
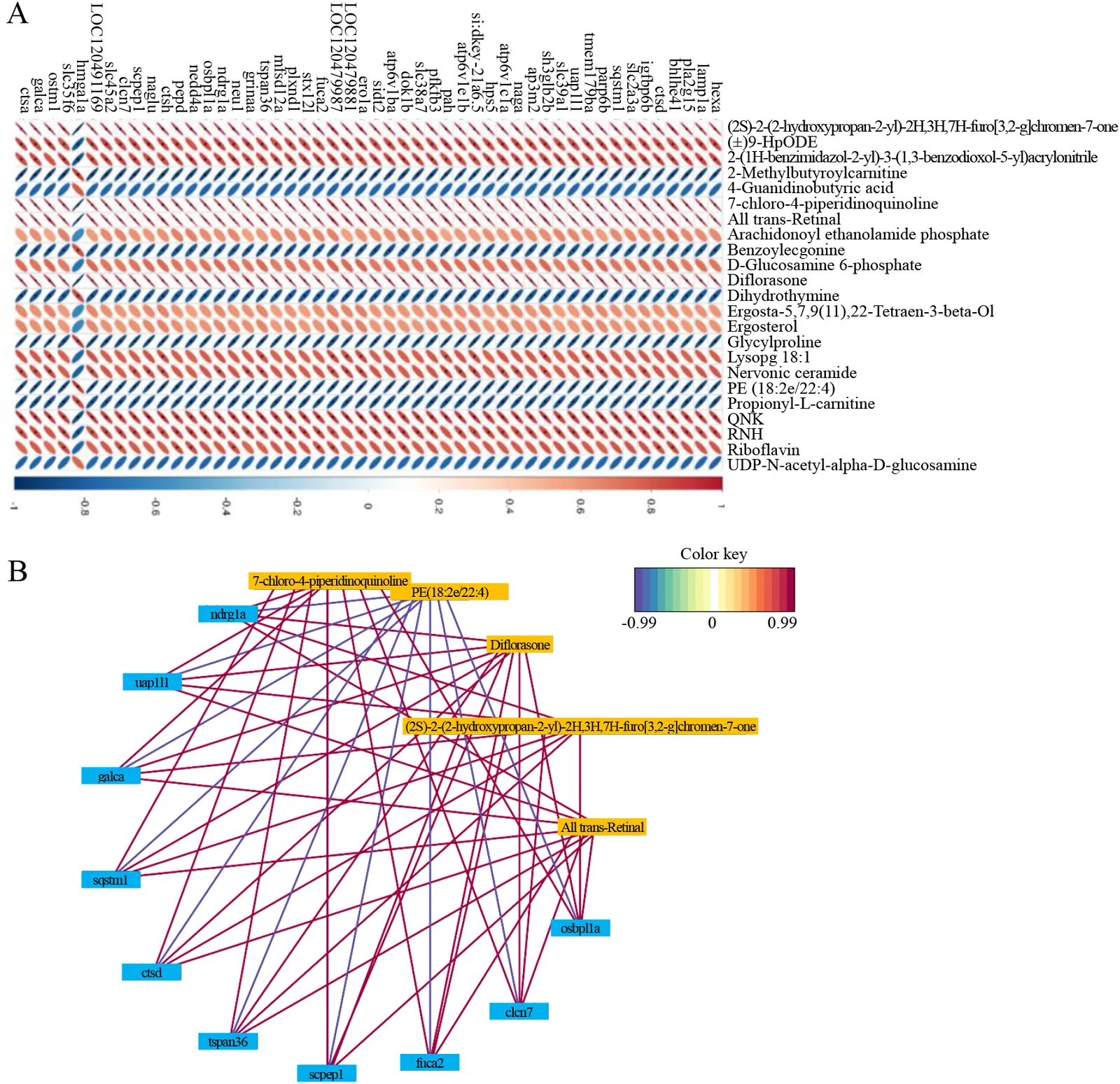
Integrated correlation analysis of transcriptomic and metabolomic data following chloroquine treatment. **(A)** Correlation heatmap showing the associations between differentially expressed genes and differential metabolites. Red indicates positive correlations, whereas blue indicates negative correlations. **(B)** Correlation network showing associations between DEGs and differential metabolites following chloroquine treatment. Nodes represent genes or metabolites, and edges represent significant correlations.

Based on the correlations identified between DEGs and differential metabolites, joint KEGG enrichment analysis was further performed to characterize the metabolic pathways associated with the coordinated transcriptomic and metabolomic changes following chloroquine treatment. Five pathways were identified, including amino sugar and nucleotide sugar metabolism, steroid biosynthesis, riboflavin metabolism, alanine, aspartate and glutamate metabolism, and arginine and proline metabolism (**Figure 11**). Among these pathways, amino sugar and nucleotide sugar metabolism showed the most significant enrichment. Steroid biosynthesis and riboflavin metabolism also showed relatively high enrichment ratios, while alanine, aspartate and glutamate metabolism and arginine and proline metabolism exhibited lower enrichment ratios. These results indicate that the transcriptional and metabolic changes following chloroquine treatment were associated with alterations in multiple metabolic pathways, particularly amino sugar and nucleotide sugar metabolism.

**Figure 11:**
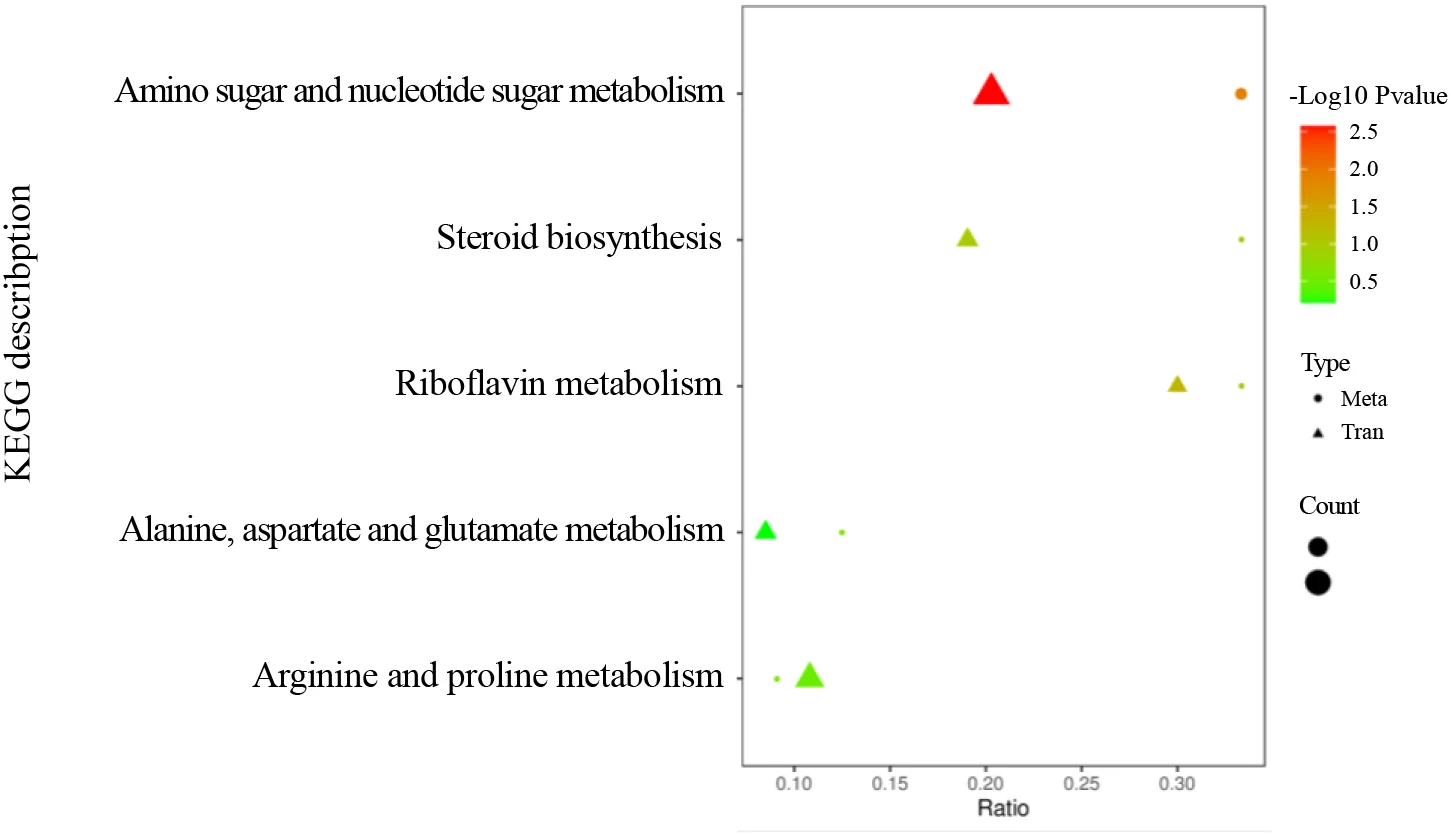
Joint KEGG enrichment analysis of transcriptomic and metabolomic data following chloroquine treatment. The dot plot shows KEGG pathways jointly enriched by differentially expressed genes and differential metabolites identified in the integrated analysis. The x-axis represents the enrichment ratio, the dot size represents the number of DEGs or differential metabolites associated with each pathway, and color indicates the enrichment significance.

### Effects of selected differential metabolites on SVCV infection

To validate the potential antiviral effects of differential metabolites, diflorasone and all-trans-retinal were selected for further experimental analysis. qPCR results showed that both metabolites reduced SVCV-G expression in a concentration-dependent manner. Compared with the SVCV-infected control group, diflorasone treatment significantly decreased SVCV-G expression at 0.5 and 2 μM, while all-trans-retinal significantly reduced SVCV-G expression at 2, 5, and 10 μM (**Figures 12A, B**). Consistently, crystal violet staining showed that treatment with diflorasone or all-trans-retinal attenuated SVCV-induced cytopathic effects. With increasing concentrations, greater numbers of cells remained attached and retained stronger crystal violet staining, indicating reduced virus-induced cell damage (**Figures 12C, D**).

**Figure 12:**
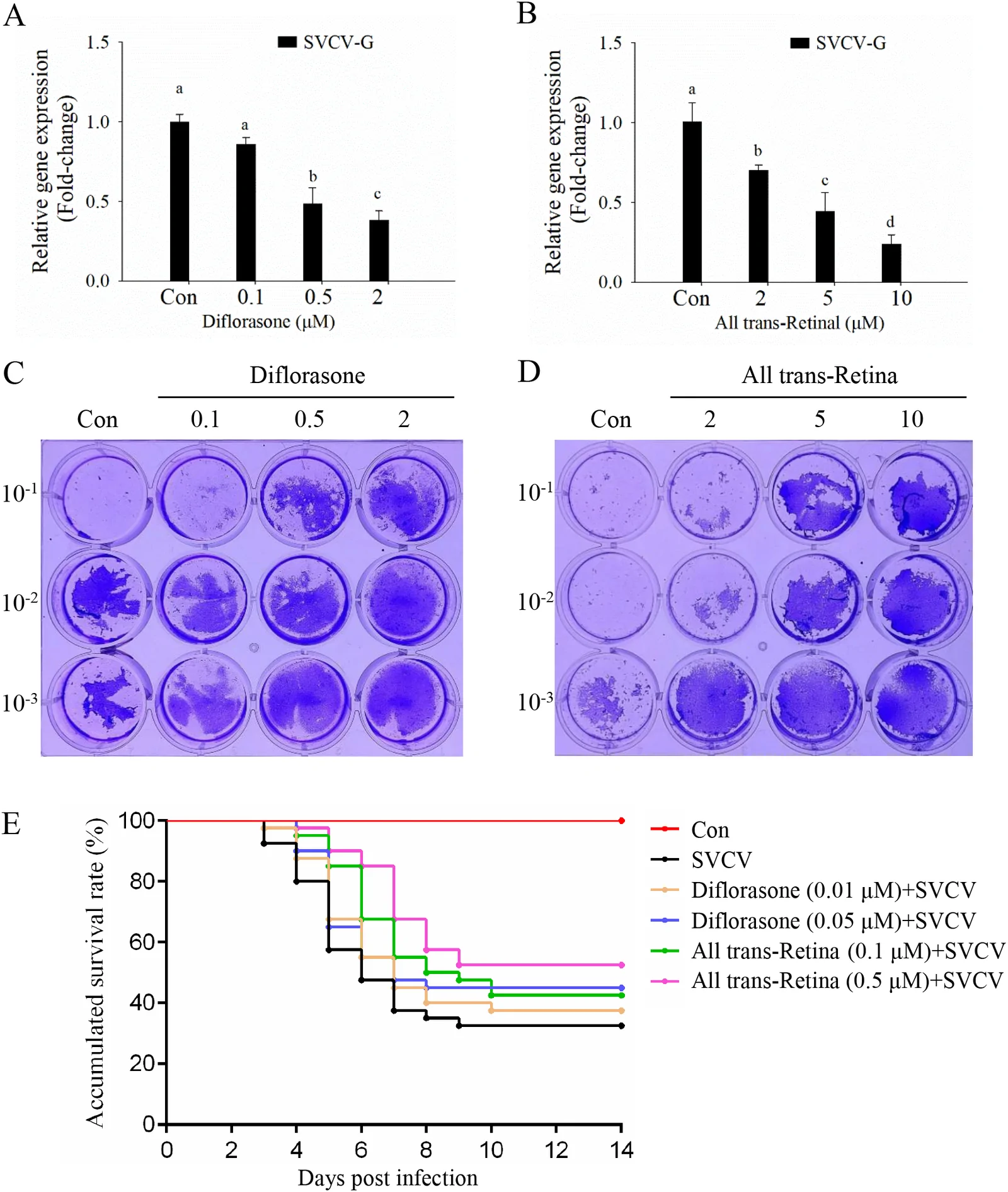
Effects of diflorasone and all-trans-retinal on SVCV infection. **(A, B)** qPCR analysis of SVCV-G expression in SVCV-infected cells treated with different concentrations of diflorasone **(A)** or all-trans-retinal **(B)**. **(C, D)** Crystal violet staining showing cytopathic effects in SVCV-infected cells treated with diflorasone **(C)** or all-trans-retinal **(D)**. **(E)** Cumulative survival rate after SVCV infection with or without diflorasone or all-trans-retinal treatment. Different lowercase letters indicate significant differences.

The protective effects of diflorasone and all-trans-retinal against SVCV infection were further evaluated in a zebrafish infection model. Compared with the control group, SVCV infection resulted in a marked reduction in cumulative survival. In contrast, zebrafish pretreated with diflorasone or all-trans-retinal exhibited improved survival following SVCV challenge. Among the tested treatments, the high-dose all-trans-retinal group exhibited the highest cumulative survival during the observation period (**Figure 12E**). These findings indicate that diflorasone and all-trans-retinal exert protective effects against SVCV infection both *in vitro* and *in vivo*, with reduced viral gene expression and cytopathic damage in EPC cells and improved survival in infected zebrafish.

## Discussion

Autophagy is a conserved intracellular degradation pathway involved in cellular homeostasis, innate immune regulation, and host responses to infection. During viral infection, autophagy can either restrict viral replication through degradation of viral components and modulation of immune signaling or be exploited by viruses to provide membranes, nutrients, and intracellular conditions favorable for replication (7, 19). Similar context-dependent effects have been reported in aquatic virus–host systems (9). Previous studies demonstrated that SVCV induces autophagy in EPC cells and that autophagy contributes to viral replication (20). In the present study, SVCV infection induced multiple autophagy-related changes, including increased autophagosome formation in the spleen of common carp, enhanced LC3 puncta formation, increased LC3-I to LC3-II conversion, decreased p62 levels, and enhanced lysosomal staining in EPC cells. Importantly, the tandem GFP-mCherry-LC3 reporter revealed increased red puncta after SVCV infection, indicating enhanced autolysosome formation. In contrast, chloroquine caused accumulation of yellow puncta, consistent with impairment of late-stage autophagic flux. Thus, in addition to confirming autophagy induction, our results provide evidence that SVCV affects the dynamic autophagic flux in EPC cells (5, 21, 22).

Pharmacological and genetic experiments further supported a positive association between autophagy and SVCV infection. Rapamycin increased SVCV-G and SVCV-I expression and aggravated SVCV-induced cytopathic effects, whereas chloroquine reduced viral gene expression and alleviated cellular damage. Consistently, ATG5 knockdown significantly reduced SVCV-G and SVCV-I expression, providing additional genetic evidence for the involvement of autophagy in SVCV infection. These findings suggest that SVCV benefits from functional autophagy rather than merely inducing autophagosome accumulation. A pro-viral role of autophagy has also been reported for several RNA viruses, including dengue virus, hepatitis C virus, and echovirus, which exploit autophagy-related membranes or signaling processes to facilitate replication (23–25). Therefore, the SVCV–EPC cell system appears to represent another virus–host interaction in which autophagy favors viral infection.

Chloroquine is widely used as a late-stage autophagy inhibitor because it accumulates in acidic organelles and disrupts lysosome-dependent degradation. It has also been shown to impair autophagosome–lysosome fusion (12). In the present study, chloroquine caused accumulation of yellow puncta in the tandem fluorescent LC3 assay and simultaneously reduced SVCV gene expression and virus-induced cytopathic damage. Together with the opposite effects observed after rapamycin treatment and the results of ATG5 knockdown, these observations suggest that disruption of the autophagy–lysosome pathway is associated with reduced SVCV replication. Similar inhibitory effects of chloroquine have been reported in several viral infection models. For instance, chloroquine inhibited hepatitis C virus replication by interfering with autophagy-dependent processes, and it also suppressed Kaposi’s sarcoma-associated herpesvirus lytic replication through disruption of mTOR and p38-MAPK activation (26, 27). However, chloroquine has multiple cellular effects beyond autophagy inhibition. Therefore, its antiviral activity should not be attributed exclusively to blockade of autophagic flux, and additional chloroquine-responsive pathways may contribute to the observed phenotype.

Transcriptomic analysis revealed broad host transcriptional remodeling after chloroquine treatment. Upregulated DEGs were particularly associated with lysosome, phagosome, autophagy, V-type ATPase, ion transport, oxidative phosphorylation, sphingolipid metabolism, and mTOR-related pathways, whereas downregulated genes were enriched in cell-cycle, DNA replication, DNA repair, and nucleotide metabolism-related processes. Because chloroquine disrupts lysosomal function, the induction of lysosome- and autophagy-related genes may represent a compensatory transcriptional response to lysosomal stress, potentially involving regulatory programs such as the TFEB/TFE3-CLEAR network (28–31). Meanwhile, suppression of cell-cycle and biosynthetic pathways may reduce the metabolic and biosynthetic capacity of host cells and thereby create an intracellular environment less favorable for viral infection (32, 33). Nevertheless, these enrichment patterns represent associations and do not establish that individual pathways directly mediate the antiviral effect of chloroquine.

Metabolomic and integrated multi-omics analyses further indicated extensive metabolic remodeling following chloroquine treatment. Differentially expressed genes and differential metabolites were particularly associated with amino sugar and nucleotide sugar metabolism, steroid and lipid metabolism, riboflavin metabolism, and several amino acid metabolic pathways. These metabolic processes are closely connected to membrane composition, energy metabolism, redox homeostasis, glycosylation, and nutrient recycling, all of which can influence viral infection (34–38). Amino sugar and nucleotide sugar metabolism was particularly prominent in the joint analysis. Because nucleotide sugars provide substrates for glycosylation, alterations in this pathway could potentially affect host glycosylation processes or maturation of viral glycoproteins. SVCV is an enveloped rhabdovirus whose surface glycoprotein mediates receptor interaction and low-pH-dependent membrane fusion (39), and glycosylation is known to influence the folding, stability, and infectivity of glycoproteins in many enveloped viruses (40). However, whether chloroquine-mediated metabolic remodeling directly alters SVCV glycoprotein processing remains to be experimentally determined. Among the differential metabolites, diflorasone and all-trans-retinal were selected for functional validation. Both compounds reduced SVCV-G expression, attenuated SVCV-induced cytopathic effects, and improved survival following SVCV challenge. All-trans-retinal is a vitamin A-related metabolite, and retinoid metabolism has been implicated in immune regulation, epithelial homeostasis, oxidative stress control, and antiviral responses (41, 42). Its protective effect may therefore involve retinoid-associated immune or metabolic pathways. Diflorasone is a corticosteroid-related compound and may influence inflammatory or stress-related cellular responses. Nevertheless, the present experiments demonstrate protective associations rather than defining their molecular targets. Further studies are required to clarify the mechanisms by which these metabolites influence SVCV infection and to evaluate their biological safety and applicability in fish.

Several limitations of the present study should be acknowledged. First, although pharmacological modulation with rapamycin and chloroquine and genetic knockdown of ATG5 support the involvement of autophagy in SVCV infection, genetic validation was limited to a single autophagy-related gene. Additional knockout, rescue, or complementary experiments targeting ATG5 and other core autophagy genes would provide stronger mechanistic evidence. Second, viral infection was mainly evaluated by viral gene expression and cytopathic effects, whereas infectious progeny virus was not directly quantified by TCID50 or plaque assays. Therefore, the present findings should primarily be interpreted as changes in viral gene expression and virus-induced cellular damage. Taken together, our results support an important role for the autophagy–lysosome pathway in SVCV infection and suggest that chloroquine-associated transcriptional and metabolic remodeling may contribute to an intracellular environment unfavorable for SVCV infection.

## Conclusion

In conclusion, SVCV infection activates autophagic flux in common carp and EPC cells, while chloroquine suppresses SVCV gene expression and cytopathic effects while blocking late-stage autophagy. Chloroquine treatment induces extensive transcriptomic and metabolomic reprogramming in host cells, affecting autophagy-lysosome signaling, cell cycle progression, and multiple metabolic pathways. Functional validation confirmed that diflorasone and all-trans-retinal efficiently inhibit SVCV infection. This study elucidates the crosstalk between autophagic flux, host metabolic remodeling and SVCV propagation, offering novel autophagy-associated metabolic targets for antiviral strategies in aquaculture.

## Data Availability

The datasets presented in this study can be found in online repositories. The names of the repository/repositories and accession number(s) can be found below: https://www.ncbi.nlm.nih.gov/, PRJNA1482244.
