# Supplementary material for: Chloroquine inhibits Spring viremia of carp virus replication by modulating autophagy and metabolic pathways
Source: Front Immunol. 2026 Oct 7;17:1920055. doi: 10.3389/fimmu.2026.1920055 (PMC13683750; doi:10.3389/fimmu.2026.1920055)
Supplement: Supplementary file 1 [file Table1.docx]

**Table S1. Primers used in this study.**

| Primers | Sequences (5’—3’) | Purpose |
| --- | --- | --- |
| siATG5 | CUGUGAUAGAAGCACAUUU | siRNA |
| SVCV-G-F | CGACCTGGATTAGACTTG | qRT-PCR |
| SVCV-G-R | AATGTTCCGTTTCTCACT |  |
| SVCV-I-F | GCCCACTTTGCATCCAGTCC |  |
| SVCV-I-R | GAGATGCCACAGACTCCTCC |  |
| ATG5-F | TGACAGACCGTTCATCCAA |  |
| ATG5-R | GGCTCATCTTCCGCACATA |  |
| qβ-actin-F | TGCAAAGCCGGATTCGCTGG |  |
| qβ-actin-R | AGTTGGTGACAATACCGTGC |  |
