# Supplementary material for: Chloroquine inhibits Spring viremia of carp virus replication by modulating autophagy and metabolic pathways
Source: Front Immunol. 2026 Oct 7;17:1920055. doi: 10.3389/fimmu.2026.1920055 (PMC13683750; doi:10.3389/fimmu.2026.1920055)
Supplement: Supplementary file 2 [file Table2.docx]

Table 2. Summary of sequencing data in the study.

| sample | Raw reads | Clean reads | Clean bases | Q20(%) | Q30(%) | GC(%) |
| --- | --- | --- | --- | --- | --- | --- |
| Ct1 | 43109494 | 42345464 | 6.35G | 97.96 | 94.14 | 47.09 |
| Ct2 | 41275924 | 40498090 | 6.07G | 98.09 | 94.50 | 45.72 |
| Ct3 | 43658260 | 43000412 | 6.45G | 98.16 | 94.64 | 46.77 |
| Tt1 | 44096748 | 43504630 | 6.53G | 98.05 | 94.42 | 46.91 |
| Tt2 | 44163486 | 43541274 | 6.53G | 98.12 | 94.49 | 46.84 |
| Tt3 | 43665468 | 43030156 | 6.45G | 98.20 | 94.73 | 46.57 |
